# Attentiveness and Fidgeting While Using a Stand-Biased Desk in Elementary School Children

**DOI:** 10.3390/ijerph17113976

**Published:** 2020-06-04

**Authors:** Ann M. Swartz, Nathan R. Tokarek, Scott J. Strath, Krista M. Lisdahl, Chi C. Cho

**Affiliations:** 1 Department of Kinesiology, University of Wisconsin-Milwaukee, Milwaukee, WI 53201, USA; ntokarek@uwm.edu (N.R.T.); sstrath@uwm.edu (S.J.S.); 2 Center for Aging and Translational Research, University of Wisconsin-Milwaukee, Milwaukee, WI 53201, USA; chocc@uwm.edu; 3 Department of Psychology, University of Wisconsin-Milwaukee, Milwaukee, WI 53201, USA; medinak@uwm.edu

**Keywords:** actigraphy, children, control group, sedentary lifestyle, standing desk

## Abstract

Standing desks are a viable option to decrease sedentary time in the classroom. However, it is important that standing desks are not detrimental to classroom behavior or learning. The purpose of this study was to evaluate the impact of stand-biased desks on fidgeting and attentiveness. Ninety-seven students in grades 3, 4, and 6 (ages 8–12 years) volunteered to participate in this study. The intervention employed a within-classroom crossover design, with teacher-determined allocation for seating within each classroom and included the replacement of one-half of the traditional sitting desks with stand-biased desks. Direct observation of student’s attentive and fidgeting behaviors occurred at three assessment periods, at baseline when all students were in a sitting desk condition and at the end of each nine-week intervention. Stand-biased desks did not influence fidgeting behavior, but did have an impact on attentive behavior. Students that were less attentive at baseline had a 40–80% increase incidence rate in non-attentive behavior while in the traditional desk as compared to the stand-biased desk after the intervention. While fidgeting and non-attentive episodes (*p* = 0.034) were significantly related, the type of desk did not significantly moderate this relationship (*p* = 0.810). Standing desks can be incorporated into the classroom without negatively influencing classroom behavior.

## 1. Introduction

The acute and long-term negative consequences of engagement in significant sedentary behavior on cardio-metabolic health, academic achievement, and emotional health of children has garnered much attention recently [1,2]. Studies have shown that habits developed in childhood are likely to carry into adulthood. For instance, children who engage in high amounts of sedentary behavior are likely to also engage in high amounts of sedentary behavior as an adult [3]. Therefore, many interventions to reduce sedentary time in children have been proposed and continue to be tested. One area where high levels of sedentary behavior occur is within school [4]. Recent reports based on objective assessments have shown that children spend approximately 70% of classroom time sitting and over 60% of school time sitting [5,6,7]. If U.S. students, for instance, spend seven hours in school, this results in 4.2 h of sedentary behavior daily. Therefore, school-based interventions have been explored as a public health approach to reduce sedentary time in children. However, because academic learning is a priority, it is imperative that interventions designed to reduce sedentary behavior in schools do not detract from academic learning.

It is well established that children are more attentive in the classroom, are better citizens of the classroom, and perform just as well or better from an academic standpoint after they have engaged in physical activity during recess or physical education [8,9,10]. Because more and higher academic stressors are being placed on schools, recess and/or physical educational opportunities for students are often reduced to create more time for learning. As a result, researchers have been exploring in-class opportunities for increasing physical activity and reducing sedentary behavior. Studies evaluating classroom-based physical activity movement breaks have shown positive impacts for both on- and off-task behavior [11,12]. Four to 10 min physical activity breaks within the classroom have been shown to reduce off-task behavior [12] and improve on-task behavior [11] in the classroom. However, these activities do require that teachers incorporate physical activity breaks into their lesson plans for the day and additional time may be required to prepare for the physical activity and to get students back to their lesson afterwards, thereby increasing the time commitment. Therefore, identifying strategies to incorporate movement and reduce sedentary behavior without taking time away from academic learning may be more desirable for both teachers and administrators.

Standing desks or stand-biased desks (desks that are accompanied by a stool to allow for sitting if needed) are a viable option to decrease sedentary time and increase physical activity in the classroom. These desks have become a recognized tool to positively influence classroom physical activity and sitting time, especially for the more sedentary students [6,13,14,15,16,17,18,19]. Despite the positive impacts on movement, it is important that standing desks are not detrimental to classroom behavior or learning if they are to become an accepted part of the classroom. Depending on how concentration or attention was measured (teacher observation, self-report, or researcher observation) and the age of the child, the effect of standing on ability to focus is not clear. Studies have shown an increase, or no change, in academic performance while using a standing desk [15,20,21,22]. In a study of sixth graders, Koepp et al. [21] found that the use of standing desks in the classroom did not affect concentration and therefore was not detrimental to learning. In another study, Dornhecker and colleagues [20] found an improvement in 2nd–4th grade academic engagement (measured by the teacher using the Behaviour Observations of Students in Schools tool [23]) after the fall but no effect in the spring compared to the control group [20]. In an older cohort of students, Sudholz et al. [18] reported that about one-third of 7th, 9th, and 10th graders reported difficulties paying attention and were distracted while using the standing desks [24]. Finally, Mehta and colleagues [22] showed that stand-biased desk use was associated with significant improvements in executive function and working memory capabilities in high school students [22]. Therefore, it appears that the effect of standing desks, as a means to increase movement and decrease sedentary behavior, on student focus in the classroom has not been fully elucidated.

Fidgeting has been used as an indication of a student’s attention [25,26]. Research is lacking on whether stand-biased desks increase or decrease the amount of fidgeting that a child engages in and how that fidgeting will affect the relationship between standing and attention. Fidgeting is the act of making small movements that are not necessarily for a particular purpose; rather the movements are a sign of restlessness, nervousness, or impatience. Fidgeting can be distracting for other children and the teacher, and it can be an indication that a student is transitioning into inattention. However, studies have shown that fidgeting can improve the task performance [27]. Therefore, it is important to consider fidgeting when examining the impact of a stand-biased desk on attentiveness.

The purpose of this study is to evaluate the impact of stand-biased desk on fidgeting and attentiveness. This study builds on previously published studies by examining both attentiveness and fidgeting while using a standing desk in multiple elementary school classrooms and a range of grades. It is hypothesized that the ability to stand and move in place, or fidget, during the day will not have a negative effect on attentiveness, thus rendering a plausible approach to increase movement, decrease sedentary behavior, while not effecting academic delivery and learning.

## 2. Materials and Methods

### 2.1. Participants and Study Overview

All 3/4 multi-age, 4th grade and 6th grade classrooms within the school and their respective teachers volunteered to participate in this study. Students, parents, and teachers in 3/4 multi-age, 4th grade and 6th grade classrooms were recruited during Registration Day for the school district through informational handouts. Interested participants attended an informational presentation during the school and classroom orientation, where all study procedures were explained and any questions answered. Willing participants were consented (parents and teachers) and assented (children) by the second week of the school year. The university institutional review board (IRB #17.019) approved all procedures.

**Figure 1:**
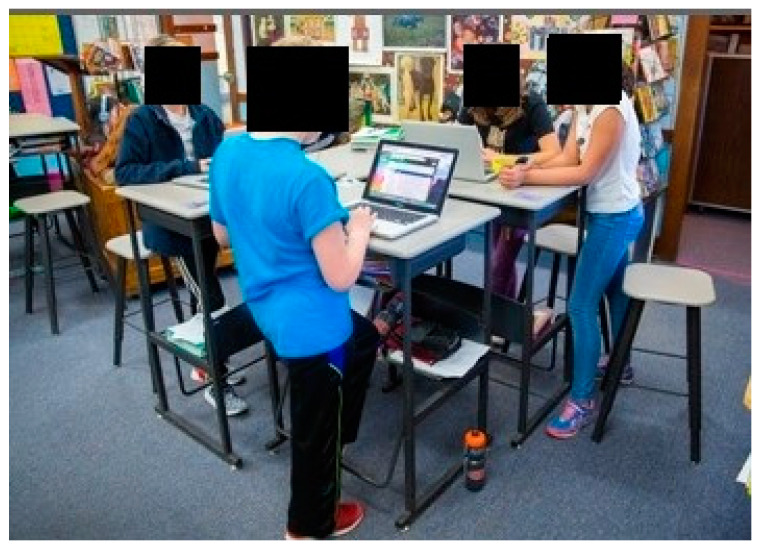
Students in the classroom at stand-biased desks.

This study was part of a larger research project where methods have been explained in detail elsewhere [13]. Briefly, this study was designed to evaluate the effect of standing during class time on a child’s ability to focus on schoolwork. The intervention included the replacement of one-half of the traditional sitting desks in the classroom with stand-biased desks. Teachers were asked to encourage students assigned to the stand-biased desks to stand in the classroom, however, no further adjustments to the classroom or curriculum were implemented. To address this purpose, this study employed a within-classroom crossover design, with teacher-determined allocation for seating within each classroom. All students, prior to study enrollment, were assigned to one of two seating options: traditional sitting desks (50% of the students) or stand-biased desks (50% of the students). The stand-biased desk (Figure 1) was a standing desk with a fidget bar for their foot accompanied by a height-matched stool, to allow the student to sit if needed (Alpha Better Height Adjustable Student Desk with Book Box, Safco Products Company, New Hope, MN). Stand-biased desks were placed in the back of the classroom, so as not to obstruct the vision of students in the traditional sitting desks.

All desks were in seated positions during baseline assessments. After baseline assessments were complete, stand-biased desks were raised to the appropriate height for each child. Students using the stand-biased desks (Stand-Sit group) used a stand-biased desk in their classroom for nine weeks during the fall (September) to winter (December) period, and then used a sitting desk for nine weeks in the winter (January) to spring (April) period. Students using a sitting desk at the start of the intervention (Sit-Stand group) used a sitting desk in their classroom for nine weeks (fall/September to winter/December), then used a stand-biased desk for nine weeks (winter/January to spring/April). The within-classroom design was selected to minimize the influence of different teachers on classroom behavior and to ensure that students remained in the desk type (stand-biased or traditional) when switching classrooms throughout the day [28].

Assessments occurred at baseline (September) and at the completion of each nine-week period (Post I/December, and Post II/April), for three assessment periods. Trained personnel completed all assessments. Assessments included direct observation of students’ behavior in the classroom. Additionally, anthropometric measurements of height, weight, distance from floor to elbow while standing (to determine appropriate height to set stand-biased desk) were recorded at baseline.

### 2.2. Direct Observation of Fidgeting & Attentiveness

Trained researchers (*n* = 3) were assigned to observe seven classrooms: observer 1 was assigned three classrooms, observer 2 was assigned two classrooms, and observer 3 was assigned two classrooms. The trained researchers observed the same classrooms at each assessment period. Each classroom was observed during a math lesson over a two-week period at each assessment time-point. Math lessons occurred at various times of the day but were consistent within classroom. Each student was observed on three separate occasions during each measurement period. These observations occurred randomly across two weeks (different days, different times during the math lesson). Researchers employed a focal-point sampling technique, observing a student’s activity for 5 s then recording the activity. Each observation and recording were repeated every 30 s in a 5 min observation window, resulting in a total of 10 5-s intervals of direct observation per 5-min window, and was repeated three times per student. In total, 30 5-s intervals of direct observation were obtained for each student. This observation protocol was repeated three times throughout the study: baseline, post assessment I, and post assessment II. Students and teachers were not aware of which students were being observed at any given time. Recording of data was signaled by a pre-recorded audio cue via earbuds, indicating when to start observing.

The trained observer recorded the activities of the participant using a purposefully built direct observation platform (Noldus Observer XT 13). Specifically, the trained observer recorded posture and movement, attentiveness, and fidgeting as seen in Table 1. On-task behavior/attentiveness was “defined as any behavior in which a student is attentive to the teacher or actively engaged in the appropriate task, as assigned by the teacher.” [29]. Off-task behavior was “defined as actions whereby a student was disengaged or distracted from the assigned task (i.e., behavior outside of the specifications “on-task” behavior).” [29]. Data was summarized by (1) summing the number of episodes out of 30, and (2) determining the average percentage of time that the child was engaging in that behavior. Specifically, the number of intervals in which the behavior occurred during the 5-min assessment period were summed, then divided by the total number of intervals and multiplied by 100 to get the average percent.

Prior to data collection, the observers attended training session to learn and practice direct observation. Observers were trained then assessed using a videotape of a classroom where students were sitting at a traditional desk, or standing at a stand-biased desk and performing the range of posture and movement, attentiveness and fidgeting. Interrater reliability was measured at each assessment period (Baseline, Post I, Post II). Interrater reliability of the trained researchers for direct observation was assessed at each data collection period and was >0.90. Inter-rater/inter-observer reliability was calculated following the procedures outlined by Mahar et al. (2006) [11].

## 3. Statistical Analysis

Continuous and categorical variables are summarized as mean ± standard deviation (SD), frequency (*n*), and percentage (%), respectively. Baseline differences between intervention groups was done using two-samples t-test, Wilcoxon rank sum test, chi-square test and Fisher’s exact test where applicable. Fidgeting and Attentiveness were summarized as counts of the number of episodes out of 30 observation windows where these events occurred. With regard to attentiveness, instead of analyzing the number of episodes where students were attentive, the number of non-attentive episodes was used as the outcome for analysis. Since these measures are counts, the negative binomial distribution was utilized to analyze the data, and the correlation between fidgeting and non-attentiveness was summarized using the Spearman rho correlation. In addition, since these measures were assessed over multiple observational periods, a generalized estimating equations (GEE) model with the negative-binomial distribution was used to determine whether the number of non-attentive episodes were significantly different when students were using a traditional desk compared to a stand-biased desk. Specifically, the main factor of interest in the model was the type of desk and the covariates included gender, grade, time, baseline measure of non-attentiveness and time-varying measure of percent of time fidgeting and sitting at each observation time. To address the aims of the study, two-way and three-way interaction between the type of desk and the covariates was also tested in the model. An alpha level of 0.05 was used to determine significant effects in the model and non-significant interaction effects were excluded from the final model. All analyses were performed using SAS 9.4 (Cary, NC, USA).

## 4. Results

### 4.1. Sample Demographics

Ninety-nine students in grades 3 (*n* = 22), 4 (*n* = 36) and 6 (*n* = 41) volunteered to participate in this study. Ninety-seven students were assented (and parents consented) to participate, of which 60% (*n* = 58) were assigned to receive the stand-biased desk during the first observation period and the remaining 40% (*n* = 39) of students used the stand-biased desk in the second observation period. Fifty-seven percent of the study population were male and 79% self-identified as White. Although the BMI percentile for the students first assigned to the stand-biased desk group was significantly higher than those assigned to the traditional desk (*p* = 0.007), the average height and weight of the students in the two groups were not significantly different. A summary of the demographic characteristics of the study sample is presented in Table 2.

### 4.2. Fidgeting and Attentive Behaviors at Baseline

On average, students were observed fidgeting 11 out of the 30 observation windows [Interquartile range (IQR): 6–15]. Interestingly, only one student was observed to not have fidgeted over the 30 observation windows and no student fidgeted in all 30-observation windows. In total, 33% of the students fidgeted less than one-fourth of the observation windows and 26% fidgeted during more than half of the observation windows, respectively. There were no differences in fidgeting behavior between boys and girls (*p* = 0.079), however, significant differences were found across grade (*p* = 0.023). Specifically, 3rd (*p* = 0.007) and 6th (*p* = 0.049) graders were found to engage in significantly more fidgeting than 4th graders. The baseline fidgeting behaviors of the students assigned to the two types of desks were not significantly different (*p* = 0.170, Table 3).

Overall, students were observed to be very attentive during their class with a median of only 3 non-attentive episodes out of the 30 observation windows [IQR: 1–6]. Further, 24% of the students were 100% attentive during the 30 observation windows and only 10 students had 10 or more non-attentive episodes. Boys were found to have significantly more non-attentive episodes than girls (*p* = 0.027), but no differences were found across grades. Finally, a significant difference in non-attentive behavior was found between students assigned to stand-biased and sitting desks. Specifically, students assigned to the stand-biased desk were found to be significantly more likely to be 100% attentive than students assigned to the traditional desks (*p* = 0.022, Table 3).

**Figure 2:**
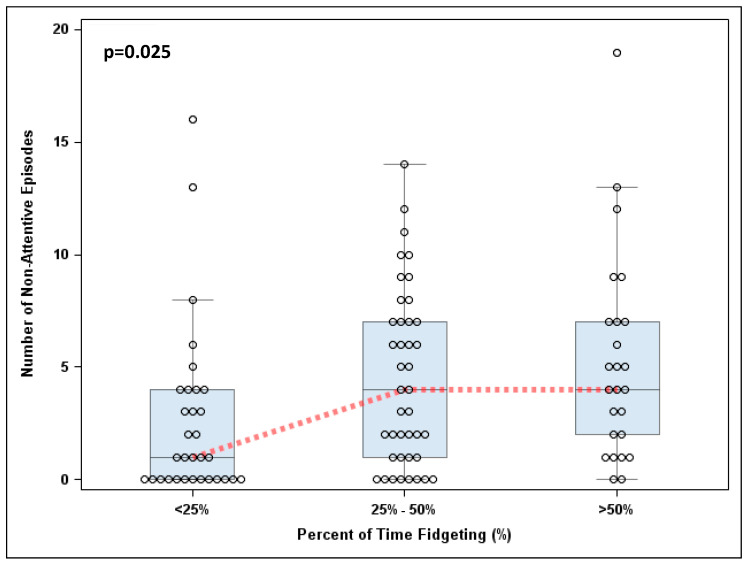
The number of non-attentive episodes by fidgeting categories at baseline.

There is a significant positive correlation between the number of fidgeting and non-attentive episodes (rho = 0.32, *p* = 0.001). However, the relationship between fidgeting and non-attentiveness is not linear (Figure 2). Specifically, the difference in the number of non-attentive episodes was found between students who fidget greater than 25% of the time compared to students who fidget less than 25% of the time (*p* = 0.005). However, there is not a significant difference in non-attentive episodes between students who fidget 25–50% of the time compared to those who fidget greater than 50% of the time (*p* = 0.528).

### 4.3. Change in Fidgeting across Time

**Figure 3:**
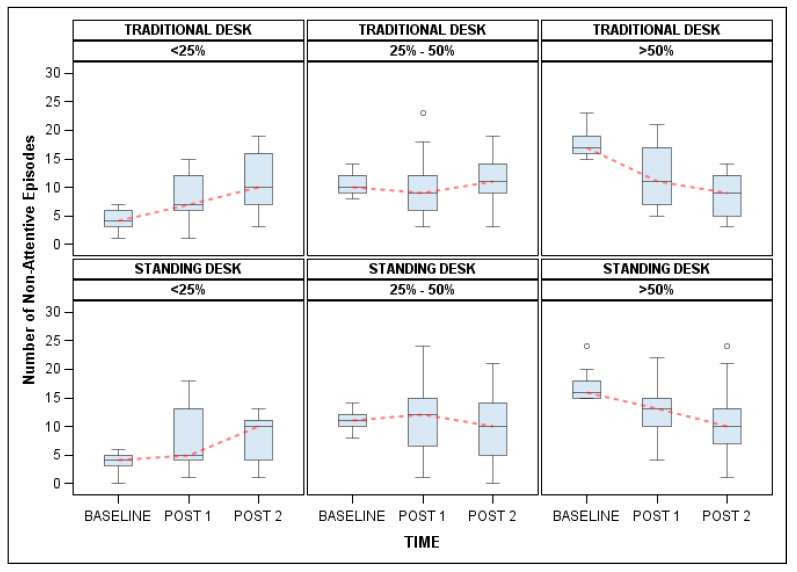
Illustration of the number of non-attentive episodes across time separated by the group assignment and baseline fidgeting behavior.

The amount of fidgeting that students engage in during class changes significantly across the study. Interestingly, the change in fidgeting is not homogeneous for each student. Particularly, the direction of change in the amount of fidgeting was influenced by the students’ baseline fidgeting behavior. Students who engaged in fidgeting less than 25% of class time at baseline tended to increase their fidgeting, whereas students who engaged in higher levels of fidgeting at baseline (>50% of the time) exhibited a decline in their fidgeting behavior (*p* < 0.001) after the nine week intervention period. It is important to note that the inverse trend relationship was found in students assigned to both intervention groups (refer to Figure 3). Since the students’ fidgeting behavior changed across time and not in a linear fashion, a time-varying three level categorical variable to summarize the percent of time students are observed fidgeting (<25%, 25–50% and >50%) was used in the modeling.

### 4.4. Change in Non-Attentive Behavior across Time

**Figure 4:**
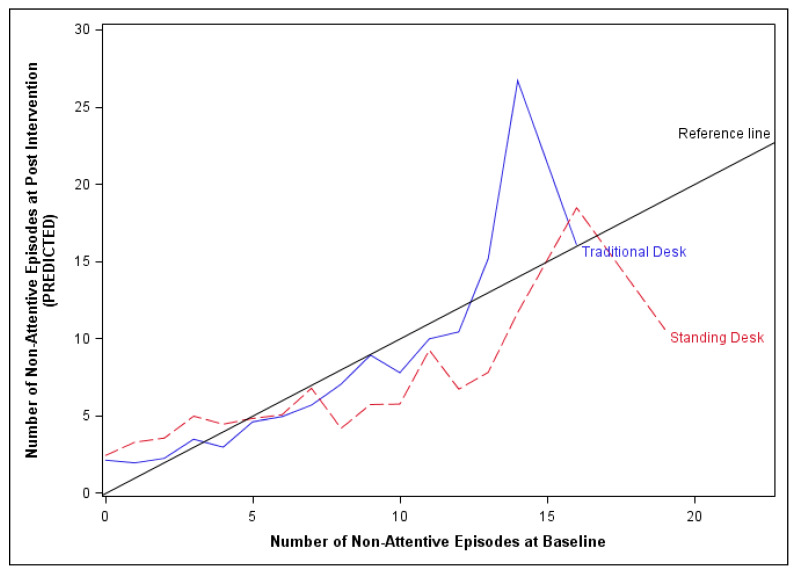
Illustration of the predicted number of non-attentive episodes at post intervention given the number of non-attentive episodes at baseline.

The results indicated that the number of non-attentive episodes was significantly different when students were using the stand-biased desk as compared to when they were using a traditional desk. However, the size and direction of the difference was dependent on the student’s baseline non-attentive behavior (*p* = 0.003, Figure 4). For example, if the student was 100% attentive (i.e., 0 of 30 non-attentive episodes) at baseline, the incidence rate of non-attentiveness was 1.44 times greater while using a stand-biased desk compared to a traditional desk (*p* = 0.040). For students whose baseline number of non-attentive episode was low (1 to 10 out of a possible 30), there was no significant difference in their incidence rate for non-attentiveness while using the stand-biased desk as compared to the traditional desk. However, for students whose baseline non-attentive behavior was between 11 to 15 episodes out of the 30 observation windows, the incidence rate of non-attentiveness increases significantly by 1.40 (11 of 30; *p* = 0.041), 1.49 (12 of 30; *p* = 0.026), 1.59 (13 or 30; *p* = 0.018), 1.69 (14 of 30; *p* = 0.013) and 1.80 (15 of 30; *p* = 0.010) times while in the traditional desk as compared to the stand-biased desk, respectively. Please note that the student’s gender, grade and amount of time sitting while using the respective desks were not significantly associated with non-attentiveness.

### 4.5. Do Stand-Biased Desks Moderate the Relationship between Fidgeting and Attention?

Results showed a significant relationship between fidgeting and non-attentive episodes (*p* = 0.034), however, the model found that the type of desk did not significantly moderate this relationship (*p* = 0.810). Although the students’ fidgeting behavior varied significantly, the relationship between fidgeting and non-attentiveness remained consistent regardless of whether the students were utilizing a stand-biased desk or a traditional desk. Specifically, after controlling for the other covariates, the incidence rates of non-attentiveness for students who fidgeted greater than 50% of the time were 1.75 and 1.63 times greater than those students who fidgeted between 25–50% (*p* = 0.001) and less than 25% of the time (*p* = 0.005), respectively. Please refer to Table 4 for a detailed result of the model.

## 5. Discussion

Results of this study show that stand-biased desks did not affect fidgeting behavior, but did influence attentive behavior. Students that were less attentive at baseline were more attentive at follow up when using the standing desk compared with the traditional desk. Further, these data showed that fidgeting and non-attentive behavior were significantly related but the type of desk being used by the student (stand-biased or traditional) did not moderate this relationship. Therefore, standing desks can be used in the classroom without negatively influencing classroom behavior.

It is important that standing desks are not detrimental to learning if they are to become an accepted part of the classroom. Depending on how concentration or attention was measured (teacher observation, self-report, or researcher observation) and the age of the child, the effect of standing on ability to focus has been positive, or shown to have no change. Studies have shown an increase or no change in academic performance while using a standing desk [15,20,21,22]. Specifically, in a small study of sixth graders, Koepp et al. [21] found that the use of standing desks in the classroom did not affect concentration and therefore was not detrimental to learning. Dornhecker [20] found an improvement in 2nd–4th grade academic engagement (measured by the teacher using the Behaviour Observations of Students in Schools tool [23]) after the fall but no effect in the spring, compared to the control group [20]. In an older cohort of students, Sudholz et al. [18] reported that about one-third of 7th, 9th, and 10th graders reported difficulties paying attention and were distracted while using the standing desks [24]. Finally, Mehta and colleagues [22] showed that stand-biased desk use was associated with significant improvements in executive function and working memory capabilities in high school students [22]. Results from this study strengthen the knowledge base for the relationship between attentiveness and standing desk use, showing that the type of desk did affect attentiveness, but was largely dependent on the baseline level of attentiveness. Those who were 100% attentive at baseline became less attentive when using the standing desk, while those who were less attentive at baseline became even less attentive over time when using a traditional sitting desk, compared with the stand-biased desk. These results need to be interpreted with caution. At baseline, 22 students were 100% attentive. At time point two and three, when they were in the traditional desk, 16/22 (72.73%) remained at 100% attentive. However, when they were in the standing desk, only 11/22 (50%) were 100% attentive. Overall, at time points two and three, students in the traditional desk had an average of 0.95 non-attentive episodes out of 30 while students using the standing desk had an average of 1.45 non-attentive episodes out of 30. Therefore, while there is a statistically significant difference, there is not a practical significance. Because they started 100% attentive, there was only one way that they could change and despite the decrease in attentiveness, these students were still very attentive. The students that recorded between 11–15 non-attentive episodes at baseline performed better while in the standing desk at time points two and three. Therefore, for students who are less attentive, the standing desk seems to have positively influenced their attentive behavior. Reasons for this were not explored in this study, however the authors hypothesize that while standing, the students perceive their actions to be more easily seen by the teacher, and therefore they may try to be more attentive. Therefore, our data supports previous studies, showing that the standing desk does not negatively affect attentiveness of students while in the classroom. In fact, the standing desk may be more advantageous than traditional sitting desks for students who do not attend to the task 100% of the time.

Research has explored the relationship between fidgeting (a behavior), and their attentional state, with studies showing a positive association between fidgeting and attention [27,30]. The stand-biased desks used in this study (SAFCO) came equipped with a “fidget bar”, a bar that was about 6 inches off the ground that students were able to rest a foot on and swing that foot back and forth while standing. While teachers, at the start of the study, were dubious about the “fidget bar”, and many lined the bar with foam pipe padding, the results of the direct observation of student behavior showed that students did not engage in more, or less, fidgeting while using a standing desk rather than traditional desks. This is the first study, to our knowledge, to address the impact of desk type on fidgeting behavior in the classroom. Regardless of desk type, those who engaged in more fidgeting at baseline decreased their fidgeting behavior over time, while those who engaged in less fidgeting at baseline increased their fidgeting behavior over time. Therefore, the standing desk did not have a negative impact on fidgeting behavior.

When the school year starts, after a prolonged break (10 weeks of summer vacation in this instance), students tend to be more heterogeneous in their activity and classroom behaviors. As the school year progressed, we observed that students tend to learn and adopt the rules of the classroom, and therefore become more homogenous in their classroom behavior. This phenomenon was seen in the present data, where students started out with a larger variation in non-attentive behavior and fidgeting behavior. However, as the school year went on, students who engaged in high amounts of fidgeting or had higher numbers of non-attentive episodes tended to reduce the number of fidgeting and non-attentive episodes. Likewise, those who did not fidget much and who were attentive at the start of the year tended to begin to fidget more and become less attentive. It is not clear from the data whether fellow students, the teacher, or some other factor influenced this change in behavior. However, the data did show that standing desks did not influence the relationship between fidgeting and attention.

While this was a straightforward environmental intervention, examining how behavior will change with the introduction of a standing desk, it is likely that additional curricular or policy changes would enhance the positive effects of the standing desk in the classroom. Additionally, feedback from teachers suggested that different teachers wanted different things from the desks, which influenced their use and adoption of the desks in the classroom. For example, some teachers would have liked to have easily adjustable desks, so that some lessons could be done standing and some sitting, rather than letting the student choose to stand or sit. Further, some teachers expressed an interest in wheels on the desks to allow collaborative work among varying student groups. Therefore, future studies should explore additional interventional tools to enhance the effects of standing desks in elementary school classrooms.

This study has a number of strengths and limitations. First, the within-classroom design allowed us to examine the influence of the desk type, while keeping the teacher, fellow students, and classroom consistent. Second, direct observation of the student behaviors in the classroom, rather than self-report by student or teacher, allowed an objective measure of our outcome variables. Limitations of this study included a primarily White student population, with generally well educated parents, many of whom used a standing desk, or were aware of what a standing desk was and the benefits of using one, therefore, there may have been positive bias towards the use of desks from parents. Additionally, given the population, this data may not be transferrable to other populations. Finally, replacing traditional desks with standing desks is a costly endeavor. At a time where funding for education is stressed, replacing traditional sitting desks that are functional may not be a high priority for schools.

## 6. Conclusions

Standing desks can be incorporated into the classroom without negatively influencing classroom behavior. Integration of stand-biased desks did not increase fidgeting in this population of students. Further, for those students who were not fully attentive, the use of a standing desk may provide some benefit. Therefore, as we look for solutions to decrease sedentary and sitting behaviors in children, and help them to build active habits and behaviors that can be sustained throughout their lives, schools can consider the implementation of standing desks in the classroom.

## Figures and Tables

**Table 1:** Description of Direct Observation.

Behavior	Characterization of Behavior
Posture and Movement	Standing Sitting Walking normal Walking fast
Attentiveness	On task Off task
Fidgeting	No fidgeting Upper body/hands fidgeting Lower body/feet fidgeting Both upper and lower body fidgeting

**Table 2:** Demographic Characteristics of Consented Participants at Baseline.

Characteristics	Type of Desk				*p*
	Traditional (*n* = 39)		Stand-Biased (*n* = 58)		
	Mean or *n*	SD or %	Mean or *n*	SD or %	
Age (Year)	10.2	1.4	10.3	1.4	0.715
Height (cm)	149.8	44.5	143.6	11.1	0.405
Weight (kg)	38.9	10.7	35.8	9.2	0.126
BMI Percentile *	65.3	47.6–89.8	39.9	23.3–70.8	0.007
Male	23	59.0	33	56.9	0.839
Grade					
3rd	10	25.6	11	19.0	0.694
4th	13	33.3	23	39.7	
6th	16	41.0	24	41.4	
Sitting at Desk (% of total time observed)					0.260
100%	18	46.15	24	42.11	
81–90%	16	41.03	18	31.58	
<80%	5	12.82	15	26.32	

Note. * Presented as median and inter-quartile range because the distribution was skewed.

**Table 3:** Fidgeting and non-attentive episodes at baseline.

Characteristics	Type of Desk				*p*
	Traditional		Stand-Biased		
	(*n* = 39)		(*n* = 58)		
	Mean or *n*	SD or %	Mean or *n*	SD or %	
Total number of fidgeting episodes *	9.00	5–14	11.00	6–15	0.138
Fidgeting (% of total time observed)					0.170
<25%	17	43.59	15	26.32	
25–50%	15	38.46	25	43.86	
>50%	7	17.95	17	29.82	
Total number of non-attentive episodes *	3.00	1–6	3.00	0–7	0.825
Non-attentiveness (% of total time observed)					0.022
<0%	4	10.26	19	33.33	
<10%	13	33.33	9	15.79	
10–20%	11	28.21	10	17.54	
>20%	11	28.21	19	33.33	

Note. Each observation and recording was repeated for 5-s every 30-s in a 5 min observation window, resulting in a total of 50 s direct observation, and was repeated three times per student at baseline, resulting in 30 observation events for a total of 150 s. * Presented as median and inter-quartile range because the distribution was skewed.

**Table 4:** Results from negative binomial model.

Effects	Estimate	SE	*p*
Intercept	−2.0977	0.2085	<0.001
Baseline Non-Attentive	0.1415	0.0203	<0.001
Fidgeting (REF: >50% of the time)			0.034
25–50% of the time Fidgeting	−0.4895	0.1753	0.005
<25% of the time Fidgeting	−0.5624	0.1750	0.001
Observation Time (REF: Post II)	−0.3031	0.1333	0.023
Stand-biased desk (REF: Traditional Desk)	0.3608	0.1756	0.040
Baseline Non-Attentive x Stand-biased desk	−0.0633	0.0213	0.003
